# Large Animal Models of Inherited Retinal Degenerations: A Review

**DOI:** 10.3390/cells9040882

**Published:** 2020-04-03

**Authors:** Paige A. Winkler, Laurence M. Occelli, Simon M. Petersen-Jones

**Affiliations:** Department of Small Animal Clinical Sciences, Veterinary Medical Center, Michigan State University, East Lansing, MI 48824, USA; winkle38@msu.edu (P.A.W.); occelli@msu.edu (L.M.O.)

**Keywords:** large animal model, inherited retinal disease, progressive retinal atrophy, retinitis pigmentosa, Leber congenital amaurosis, achromatopsia, congenital stationary night blindness

## Abstract

Studies utilizing large animal models of inherited retinal degeneration (IRD) have proven important in not only the development of translational therapeutic approaches, but also in improving our understanding of disease mechanisms. The dog is the predominant species utilized because spontaneous IRD is common in the canine pet population. Cats are also a source of spontaneous IRDs. Other large animal models with spontaneous IRDs include sheep, horses and non-human primates (NHP). The pig has also proven valuable due to the ease in which transgenic animals can be generated and work is ongoing to produce engineered models of other large animal species including NHP. These large animal models offer important advantages over the widely used laboratory rodent models. The globe size and dimensions more closely parallel those of humans and, most importantly, they have a retinal region of high cone density and denser photoreceptor packing for high acuity vision. Laboratory rodents lack such a retinal region and, as macular disease is a critical cause for vision loss in humans, having a comparable retinal region in model species is particularly important. This review will discuss several large animal models which have been used to study disease mechanisms relevant for the equivalent human IRD.

## 1. Introduction

Large animal models for inherited retinal degenerations (IRDs) have been identified within populations of dogs, cats, sheep, horses and, more recently, non-human primates (NHP). Many different IRDs have been identified in pedigree dogs, most of which mimic retinitis pigmentosa (RP) or Leber congenital amaurosis (LCA). The term “progressive retinal atrophy” (PRA) is used to describe this group of photoreceptor degenerations. In some instances, more detailed descriptive terms such as rod–cone dysplasia or progressive rod cone degeneration have been used. Common dog breeding practices have tended to bring out recessive conditions and have made the pedigree dog a rich source of models for inherited disease, including IRDs. Engineered large animal IRD models such as transgenic pigs have also been produced [1]. With advances in gene editing technologies, further models are likely to be produced in species such as pigs and NHPs and possibly even cats and dogs. The advantages of large animal models over laboratory rodent models of IRDs include the similarity in the size of the eye to that of man [2]. This is of particular importance for the development of translational therapies because it allows identical surgical delivery approaches to be used in the animal model to those that will be eventually used in human patients. NHPs are obviously close relatives to humans, making them attractive models. However, only a few spontaneous IRDs in primates have been identified [3,4], although steps are being taken to identify more animals with disease-causing mutations and to use genome editing to generate additional models with mutations in genes of importance, either for germline transmission (see [5] for a review) or somatic gene knockout [6]. Another major advantage of large animal models is the presence of a retinal region equivalent to the macula. Laboratory mice and rats are nocturnal rodents that do not have a macula equivalent. The macula is of major importance for high acuity vision and some conditions specifically or differentially affect that retinal region compared to the peripheral retina. Macular dystrophies have been associated with mutations in a number of different genes (see [7,8] for reviews), some of which have relevant large animal models which are discussed below, including Stargardt Disease (*ABCA4* mutations), Best Disease (*BEST1* mutations) and in some patients with *RDH5* mutations. Age-related macular degeneration (AMD) is a major cause of vision loss and has genetic and environmental contributors [9,10]. Screening of primate colonies for animals with lesions comparable to AMD have been performed [11] (for a review, including primate models, see Pennesi et al. [12]). The large animal model species considered here have an area centralis with high photoreceptor density, including, importantly, cones that are equivalent to the human macula [13]. While NHP also have a fovea, most of the other model species do not, although the dog has been reported to have a small concentration of cones in the center of the area centralis, referred to as a “bouquet” of cones [14].

There are several important instances where laboratory rodent engineered models fail to recapitulate the human disease; important examples including *ABCA4*-Stargardt disease, *RDH5*-retinopathy, or where the gene involved is not present in the mouse or rat genome e.g., *EYS* [15].

The disadvantages of the large animal model species tend to be cost, generation of sufficient animals due to the slower reproduction, and because of the longer lifespan (compared to laboratory rodents), the disease course may be longer.

Large animal models have also been important in therapy development. The first proof-of-concept gene augmentation therapy that eventually led to the FDA approval of the first gene therapy product, Luxturna, for the treatment of LCA due to *RPE65* mutations, was performed in a dog model [16].

**Figure 1:**
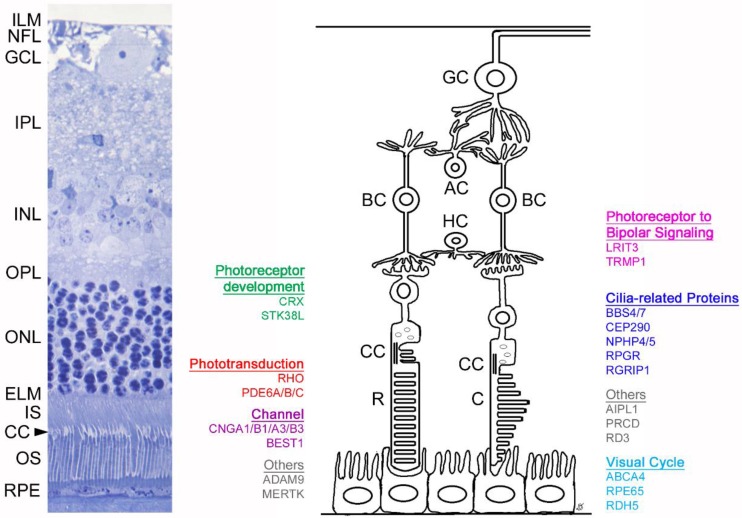
Schematic of retinal layers and associated genes discussed within this review. The left image shows a histologic section of a feline retina (with comparable anatomy to the human retina). The right panel depicts a schematic showing the genes detailed in this review and their site of expression, grouped per biological process. Inner limiting membrane (ILM), nerve fiber layer (NFL), ganglion cell layer (GCL), inner plexiform layer (IPL), inner nuclear layer (INL), outer plexifom layer (OPL), outer nuclear layer (ONL), external limiting membrane (ELM), photoreceptor inner segment (IS), connecting cilium (CC), photoreceptor outer segment (OS), retinal pigmentary epithelium (RPE). Ganglion cell (GC), amacrine cell (AC), bipolar cell (BC), horizontal cell (HC), rod (R), cone (C).

Table 1 shows a list of IRDs in large animal models and their identified mutations. Retinal layers and the genes discussed in detail within the text are shown in Figure 1. This review will cover the models in which the studies have contributed to the understanding of the mechanism of disease and/or protein structure and function in greater detail.

## 2. Mutations in Phototransduction Genes

There are spontaneously occurring large animal models with mutations in the genes of the phototransduction cascade, and rhodopsin (*RHO*) transgenic pig models have been generated. Studies of these models have contributed to the understanding of how the mutation of these genes leads to photoreceptor death.

### 2.1. RHO

Mutations in *RHO* can result in a range of phenotypes, most commonly autosomal dominant RP (adRP), but also autosomal recessive RP (arRP) and congenital stationary night blindness (CSNB). Animal models and in vitro studies have allowed *RHO* mutations to be divided into seven classes (see [17] for a recent review).

#### 2.1.1. Dog Model

A spontaneous dog model of *RHO* adRP has been identified [18]. The c.11C>G, p.Thr4Arg mutant dog (*Rho^T4R^*) develops a retinal degeneration that is greatly exacerbated by light exposure [19]. The phenotype of this dog model closely resembles that of the human p.Thr4Lys *RHO* mutation which results in dominant RP [20]. Studies of the *Rho^T4R^* dog have advanced the understanding of the disease mechanism underlying class four *RHO* mutations (those with altered post-translational modification and stability [17]).

RHO has seven transmembrane loops with intradiscal and cytoplasmic loops and in the dark-adapted state is combined with the chromophore 11-cis-retinal. The N-terminal of the protein, which is affected by the p.Thr4Lys mutation, is positioned within the lumen of the outer segment discs and creates a “cap” over one of the extracellular loops. This cap contributes towards thermal stability and receptor activation of the protein; it also protects the chromophore to opsin protein covalent bond from hydrolysis. Important for the cap role of the N-terminal is glycosylation at N2 and N15. The p.Thr4Arg mutation interferes with glycosylation at the N2 site, altering the cap role. The monoglycosylated rhodopsin is expressed and is trafficked to the outer segment; however, it loses the chromophore faster than the normal meta-rhodopsin II and interacts poorly with the G-protein [21]. The *Rho^T4R^* dog has light dependent degeneration similar to the sector RP seen with some rhodopsin mutations. In sector RP, the inferior retina is more severely affected as this region gets more light exposure [22]. There is increasing awareness of the need to reduce light exposure to patients with certain *RHO* mutations [23]. Studies suggested that the unliganded form of the mutant opsin has a detrimental effect because of the loss of its structural integrity. Further evidence to support this was provided by cross breeding *Rho^T4R^* dogs with the *Rpe65^−/−^* dog to produce *Rho^T4R/+^ Rpe65^−/−^* dogs which lack 11-cis-retinal chromophore (due to the lack of Rpe65 function) and thus have only unliganded mutant rod opsin (i.e., have a lack of rod opsin combined with 11-cis-retinal) and show a greatly accelerated rate of retinal degeneration compared to *Rho^T4R/+^Rpe65^+/+^* dogs (see details on the *Rpe65^−/−^* dog below) [21].

#### 2.1.2. Pig Models

A number of *Rho* transgenic pigs have been generated, representing different human mutations: p.Pro23His [24], p.Pro347Leu [25] and p.Pro347Ser [26]. Studies using the p.Pro347Leu pigs showed the development of ectopic cone to rod bipolar cell synapses [27] and also interference with the cone to OFF-bipolar cell connection maturation [28]. The potential contribution of oxidative stress to cone death was demonstrated in the model [29]. The light responses of single rod photoreceptors of p.Pro347Leu and p.Pro347Ser transgenic pigs have been studied by suction pipette recording. The recording revealed protracted recovery of the photoresponse and a progressive reduction in the time to peak of the response with reduced sensitivity. This work suggests that the mutant rhodopsin reaches the outer segment and that the substitution at Pro347 interferes with inactivation of the activated form of Rho. The resulting hypothesis was that the carboxyl end of Rho may be involved in the binding of rhodopsin kinase. Mutations at Pro347 may reduce the stability of the carboxyl end attachment to rhodopsin kinase, potentially slowing phosphorylation and the subsequent binding of arrestin [30].

The p.Pro23His pig model has been used to study factors associated with cones developing dormancy and whether they can be reactivated. There are stages of retinal degeneration where the degenerating cones lose inner and outer segments. The remaining cell bodies are described as dormant cones. One hypothesis is that a lack of glucose supply to the cones as a result of loss of surrounding rods leads to cone dormancy. Experiments to either introduce rod precursors or to supply glucose to the subretinal space resulted in reactivation of the dormant cones suggesting mechanisms for the treatment of later-stage IRDs [31].

### 2.2. Phosphodiesterase 6 Genes

Mutations in genes encoding for the subunits forming the rod specific cyclic guanosine monophosphate (cGMP) phosphodiesterase (PDE) holoenzyme cause about 36,000 cases of autosomal recessive RP worldwide in humans, leading to the early onset of night blindness and retinal degeneration [32].

The rod PDE heteromeric holoenzyme has a catalytic core made of PDE6A and PDE6B subunits combined with two inhibitory gamma subunits [33]. Mutation in the gene encoding the alpha subunit of cGMP-PDE (Pde6a) causes PRA in the Cardigan Welsh corgi dog [34,35] and is a model of RP43 in humans, which accounts for 3% to 4% of recessive RP in North America [36,37]. Mutations in the gene encoding the beta subunit of cGMP-PDE (Pde6b) have been identified in a few dog breeds (see below), including the Irish setter dog [38], which is a model for RP40 in humans and represents about 3% to 5% of the recessive forms of RP [39,40]. Disease mechanisms for these canine models are detailed below.

#### 2.2.1. PDE6A

A dog model with a null mutation in the gene encoding for Pde6a has been identified. It has a relatively severe phenotype and is a model for RP43 in humans [34,35]. This form of PRA was given the name rod–cone dysplasia type 3 (*rcd3*). The frameshift mutation, c.1939delA. p.Asn616ThrfsTer39, results in the absence of Pde6a in the affected dog retina (*Pde6a^−/−^*) [35]. Western blot analysis shows the absence of all Pde6 subunits, showing the requirement of Pde6a for stability and normal trafficking of Pde6b [35]. In the absence of the Pde6 holoenzyme, the cGMP hydrolyzing activity is absent and cGMP accumulates in the rod photoreceptors [41]. Increased cGMP is a well-established cause of photoreceptor cell death, likely due to the increased influx of calcium ions into the outer segment [42], triggering apoptosis [43]. The rod outer segments fail to mature in *Pde6a^−/−^* dogs and the genetically unaffected cones have stunted outer segments, which is reflected in a reduction in cone electroretinogram (ERG) a-waves early in the disease process [35]. Following the death of rod photoreceptors, there is a progressive loss of cones, which is reflected in the declining cone ERG amplitudes which eventually become undetectable at about one year of age (SMPJ unpublished data). Adeno-associated gene therapy was able to rescue rod function and promote cone function, as well as preserve retinal morphology [41,44].

#### 2.2.2. PDE6B

The catalytic beta subunit (PDE6B) of the cGMP-PDE heteromeric holoenzyme is another essential component of the rod photoreceptor phototransduction cascade located in the outer segments [33]. Mutations in the gene encoding for Pde6b causes PRA (rod–cone dysplasia type 1, *rcd1*) in the Irish Setter dog [38,45,46] which is a model for RP40 in humans. RP40 is one of the most common autosomal recessive RPs leading to blindness in midlife in humans. As with the *Pde6a^−/−^* dog, rod photoreceptors are affected first, then cones later in the disease [47,48]. The *Pde6b^−/−^* dog phenotype has an autosomal recessive mode of inheritance and is caused by a nonsense mutation (c.2420G>A, p.Trp807Ter) [38,45]. This truncated protein would lack the C-terminal domain that is required for posttranslational processing and membrane association. The failure of phosphodiesterase activity due to a lack of function in Pde6b leads to elevated cGMP levels from an early age [47,49]. The elevation of cGMP in rods as they develop outer segments results in the halting of outer segment elongation followed by rod degeneration, starting in the central retina first, then spreading to the entire retina following the same pattern of rod maturation. The cone outer segment development is also halted and, with the loss of the rods, the genetically unaffected cone photoreceptors also progressively degenerate [46,47]. Interestingly, in contrast to the *Pde6a^−/−^* dog retina where the lack of Pde6a leads to the absence of both alpha and beta Pde6 subunits, the *Pde6b^−/−^* dog retina has a detectable Pde6a subunit in Western blot, prior to rod degeneration [38]. Therefore, it appears that the alpha subunit is necessary for the beta subunit to be maintained, while the beta subunit is not essential for the maintenance of the alpha subunit. Adeno-associated gene therapy has been shown to halt retinal degeneration in the *rcd1* dog [50]. 

Mutations in *Pde6b* have been identified in at least two other breeds of dogs (Sloughi and American Staffordshire terriers; see Table 1 for mutation information) [51,52].

#### 2.2.3. PDE6C

Recently, a NHP spontaneous achromatopsia model was identified with a missense mutation in a cone phosphodiesterase subunit gene (*Pde6c*; c.1694G>A, p.Arg565Gln) [4]. Affected animals had behavioral changes, reflecting the photophobia seen in human subjects. They also had macula changes including foveal thinning and a subtle bullseye maculopathy. In vitro studies suggested that the mutant protein was expressed and colocalized with its chaperones, Aipl1 and Pɣ. However, the mutation alters the catalytic domain, meaning that the mutant protein fails to hydrolyze cGMP.

## 3. Visual Cycle

### 3.1. ABCA4

Stargardt disease is an inherited macular dystrophy which affects one in 8,000–10,000 people. It is the most common inherited macular dystrophy and there is currently no cure. It results from mutations in the gene *ABCA4*. Mutations in *ABCA4* also result in cone–rod dystrophies and RP. ABCA4 is an ATP-dependent flippase expressed in the photoreceptor disc membrane and is necessary in the visual cycle for its transport of N-retinylidene-phosphatidylethanolamine and phosphatidylethanolamine out of the lumen into the cytoplasm [53].

A 1 bp insertion in *Abca4* was identified in a family group of Labrador retriever dogs resulting in a frameshift and premature stop codon [54]. This mutation causes a decrease in the mRNA transcript and the loss of the full-length protein. As seen in humans, there is an accumulation of lipofuscin in the RPE cells. Cone and rod photoreceptors both had abnormal function and were decreased in number in older affected dogs (10+ years). The development of a colony of these dogs as a model for therapy will have a substantial impact on the treatment of humans with Stargardt disease because mouse *Abca4^−/−^* models lack a phenotype [54,55]. The phenotype in the dog appears to be milder than that seen in human subjects, with *ABCA4* mutations reflecting species differences [56]. Early biomarkers of retinal changes in the affected dogs will facilitate the use of the *Abca4* mutant dog as a model for human Stargardt disease.

### 3.2. RPE65

Large animal models with visual cycle gene mutations include the *Rpe65^−/−^* dog. This is a model for LCA2. This model was crucial in the development of translational gene augmentation therapy which led to the first FDA-approved gene therapy product. The first animal injected with a therapeutic vector for LCA2 was an *Rpe65^−/−^* dog. Therapies were developed by three independent groups and consisted in each instance of recombinant adeno-associated virus vectors packaged with *RPE65* cDNA. The precise details of promoters and other features such as polyadenylation signals and enhancers differed between the groups. Four groups with colonies of *Rpe65^−/−^* dogs reported successful restoration of rod and cone function [16,57,58,59]. Loss of rod photoreceptors in the *Rpe65^−/−^* dog was slow and gene therapy showed ERG rescue even in middle-age [60]. Studies showed that S-cones were sensitive to the lack of normal 11-cis-retinal supply and s-cone opsin immunoreactivity was lost at an early age [61]. There were some phenotypic differences between the *Rpe65^−/−^* dog colonies, with one showing early photoreceptor degeneration in the area centralis (canine equivalent of the human macula) [62]. Trials in human subjects have not resulted in the same restoration of function shown by the dramatic improvement in ERG responses seen in dog and mouse models. A possible explanation for this species difference in therapy efficacy was provided by a comparison of the Rpe65 function of primates and dogs. This suggested that primates require a higher level of Rpe65 than dogs for the function of the visual cycle and that the current therapy might not result in adequate levels of enzymatic function in humans [63].

### 3.3. RDH5

Recently, a cat model with a mutation in another visual cycle gene, *Rdh5*, has been identified by our group [64]. Rdh5 functions to convert 11-cis-retinol to 11-cis-retinal for transport to photoreceptors for reforming the visual pigments. In humans, *RDH5* mutations cause a variety of phenotypes. Fundus albipuncatatus is the predominant phenotype [65] but a subset of patients have macular atrophy [66,67,68,69] or cone dystrophy [70]. The *Rdh5*-mutant cat promises to be a valuable model because the *Rdh5^−/−^* mouse lacks a phenotype and does not recapitulate *RDH5*-retinopathy in human patients [71]. In contrast, the cat model, similar to affected humans, shows a very delayed recovery of photoreceptor function following light exposure and recapitulates the *RDH5*-macular atrophy phenotype.

## 4. Channelopathies/Channel-Related Mutations

Mutations that affect channel protein structure and function resulting in disease are termed channelopathies. Spontaneously occurring retinal channelopathies have been identified in dogs resulting from mutations in the cyclic nucleotide-gated ion channels (CNG) of the rod and cone photoreceptors and the anion channel bestrophin 1 (BEST1).

CNG channels in the rod and cone photoreceptors are directly involved in phototransduction. The CNG channels expressed in the rod photoreceptor consist of four subunits: three CNGA1 and one CNGB1 [72,73,74]. The CNG channel in cone photoreceptors consists of CNGA3 and CNGB3 proteins, in a 3:1 or 2:2 ratio (the stoichiometry of the CNGA3/CNGB3 channel is under debate) [72]. Damaging mutations in the rod CNG channels result in RP, while mutations in cone CNG channels result in achromatopsia.

BEST1 is a homopentameric channel expressed in the retinal pigment epithelium (RPE) and involved in anion transport and intracellular calcium homeostasis [75]. Mutations in *BEST1* result in a collection of retinal diseases. Mutations in *BEST1* often result in Best Vitelliform Macular Dystrophy, but the age of onset, mode of inheritance, disease characteristics and prognosis can vary [75].

### 4.1. CNGA1

In humans, damaging mutations in *CNGA1* result in RP49 representing 1% or less of arRP cases [39]. A 4 bp deletion in *Cnga1* was identified in Shetland Sheepdogs with PRA. The mutation causes a frameshift and a premature stop codon in a highly conserved region of the protein [76].

### 4.2. CNGB1

There are three splice variants of *CNGB1* expressed in the retina, glutamic acid rich proteins (GARPs) GARP1, GARP2 and CNGB1a. The full-length protein, CNGB1a, is part of the heterotetrameric CNG channel of the rod photoreceptor [72]. A complex mutation in *Cngb1* was identified in Papillon dogs with PRA. The mutation identified in these dogs is a 6 bp insertion accompanied by a 1 bp deletion, predicted to result in a frameshift and a premature stop codon, six amino acids downstream [77]. Upon further analysis of the *Cngb1a* transcript in the *Cngb1^−/−^* dogs, it was found that the mutation led to the skipping of exon 26, resulting in a premature stop codon early in exon 27. A truncated Cngb1a protein is produced, but does not form channels with Cnga1 and remains in the inner segments of the rod photoreceptors [78]. Mutations in *CNGB1* cause RP45 in humans, which represents less than 4% of arRP cases [39].

Human RP45 patients with mutations downstream of the GARP splice variants, the mouse knockout model (*Cngb1-X26*) and the *Cngb1^−/−^* dog have similar phenotypes [78,79]. The canine model shows a slow loss of rod photoreceptors and the relative preservation of cones, particularly in the area centralis and visual streak. Cone function decreases as the disease progresses, as assessed by ERG, but cone-led vision remains normal, at least up to four years [77]. Preliminary gene therapy trials have shown that treatment at 3.5–6.5 months of age in affected dogs rescues vision and slows the progression of the disease in the treated areas [78].

Both the slow disease progression and the large treatment window (in dogs and anticipated in humans [80]) make the *Cngb1^−/−^* dog an ideal model for studying therapies and outcome measures.

### 4.3. CNGA3

About 25% of human achromatopsia cases are caused by damaging mutations in the alpha subunit of the cone CNG channel (*CNGA3*) [81]. Two canine models of achromatopsia were identified with mutations in *CNGA3*. The mutations, p.Arg424Trp and p.Val644del (R424W and V644del, respectively), provided intriguing mutation sites for in vitro testing. Modeling of these mutations lead to further insights into CNG channel gating and subunit interactions [82]. The residue R424 is located in the S6 transmembrane helix and forms a salt bridge with the residue E306 in the S4–S5 linker. Protein modeling of the canine R424W mutation found the disruption of this salt bridge plays an important role in protein folding, subunit assembly and channel gating. In vitro expression studies of the R424W mutation showed increased mislocalization of the mutant CNGA3 protein and the mutant channels did not produce cyclic nucleotide-activated currents [82].

The canine V644del mutation is in the C-terminal leucine zipper (CLZ) domain, which is involved in channel assembly and stability. The one amino acid deletion shifts all subsequent residue interactions within the domain. In vitro studies in a heterologous expression system showed evidence of mislocalization and a decrease in the total number of active channels, but some mutant channels (~60%) reached the cell membrane and had normal cyclic nucleotide-activated currents [82].

Two spontaneous sheep models of *Cnga3* achromatopsia have been identified [83]. The first identified has a nonsense mutation (p.Arg236Ter) and the second a missense mutation (p.Gly540Ser) [84]. Adeno-associated virus gene therapy was able to restore cone function in both models [84,85].

### 4.4. CNGB3

Two unique mutations were identified in the canine *Cngb3* gene—a genomic deletion that removes the entire *Cngb3* gene (*Cngb3^−/−^*) and a missense mutation (p. Asp262Asn; D262N). The missense mutation was identified in German Shorthaired Pointers, while the genomic deletion was initially found in Alaskan Malamutes and later found in other breeds [86,87]. Mutations in *CNGB3* are responsible for at least 50% of achromatopsia cases in humans, making it an ideal target for therapies [87,88].

The D262N mutation in *Cngb3* inspired investigation into the Tri-Asp motif that is conserved in CNG channels [89]. When this mutation was introduced to heterologously expressed CNGA3, it abolished homomeric channel function and was responsible for mislocalization of the protein. When the mutation was introduced in CNGB3 in coexpression studies with CNGA3, the response to cyclic nucleotides was reduced but still present and consistent with CNGA3 homomeric channel function. These experiments show the Tri-Asp motif is necessary for proper cone CNG channel formation, but raises the question why mutations in *CNGB3* result in the loss of cone function and achromatopsia when CNGA3 can form a functioning homomeric channel in the absence of CNGB3. Further work in retinal tissue and/or cone photoreceptors will be needed to elucidate this [89].

Delivery of a human *CNGB3* gene via rAAV5 vector to both *Cngb3^−/−^* and the D262N mutant dogs showed that the vector could be targeted to the medium and long wavelength (M/L) cones via a human red cone opsin promoter and the rescue was dependent on the age of the dog and the promotor, not on the mutation type [90].

### 4.5. BEST1

Best vitelliform macular dystrophy (Best disease, BVMD) has autosomal dominant inheritance and is the most common disease associated with mutations in the gene *BEST1*. Four other disease phenotypes have been described in association with *BEST1* mutations: adult onset vitelliform macular dystrophy, autosomal recessive bestrophinopathy, autosomal dominant vitreoretinochoroidopathy and retinitis pigmentosa. BVMD is characterized by at least one vitelliform lesion in the macula but can present with multiple lesions. The disease slowly progresses to degenerate the RPE and retina in the affected regions, resulting in vision impairment [75]. In dogs, a similar disease has been described, termed canine multifocal retinopathy (CMR) and is caused by mutations in the *Best1* gene. In contrast to the BVMD in humans, CMR due to *Best1* mutations is an autosomal recessive disease and has a consistent and predictable disease phenotype. This consistency and the detailed natural history of the disease lends well to measuring the outcomes of translatable therapies.

Initially, two *Best1* mutations were identified in dogs: p.Arg25Ter (Great Pyrenees and mastiff-related breeds, *cmr1*) and p.Gly161Asp (Coton de Tulear, *cmr2*), with the former resulting in a premature stop codon and, presumably, a lack of Best1 protein [91]. Analysis of additional breeds with CMR identified another breed (Lapponian herder, *cmr3*) with two deleterious mutations in exon 10, a 1 bp deletion leading to a frameshift and premature stop codon (p.Pro463fs) and a missense mutation (p.Gly489Val) [92]. Interestingly, the phenotype resulting from the three mutations in dogs is indistinguishable. The dogs present with multifocal regions of retinal separation with a pink or tan-colored subretinal fluid, which eventually leads to retinal degeneration. Using optical coherence tomography to image eyes from *cmr1/cmr1*, *cmr3/cmr3* and *cmr1/cmr3* dogs in vivo, the earliest detectable sign of the disease is at ~11 weeks of age in which there is a retinal elevation in the fovea-like region of the area centralis. As the disease progresses, this retinal elevation becomes a macrodetachment, surrounded by microdetachments. Rates of progression, detachment location and number varied, but the disease was typically localized to the more cone-rich regions of the retina [93].

Immunohistochemistry of CMR canine tissues showed a lack of RPE apical microvilli at the cone photoreceptor/RPE interface along with accumulated lipofuscin within the RPE. The loss of the RPE apical processes results in a loss of all direct contact of the cones to the RPE, severely impacting the physiological role the RPE has on retinal maintenance. It is hypothesized that the lack of RPE apical processes and subsequent weakened interphotoreceptor matrix is instrumental in the characteristic detachments and lesions observed in diseases caused by *BEST1* mutations [93,94]. Interestingly, microdetachments were identified in response to light exposure in pre-clinical CMR dogs. The detachments occurred between the photoreceptor inner/outer segments and the RPE/tapetum interface. These light-induced detachments occurred within minutes and increased in response to time of light exposure and would resolve within 24 h.

Using a rAAV2 vector delivered subretinally, CMR dogs were treated by gene augmentation with wildtype canine or human *BEST1*. Macro and microdetachments were resolved and RPE microvilli ensheathment of cone photoreceptors returned within the treatment area. This positive outcome was retained in the dogs for as long as 207 weeks post injection. The same outcome was present regardless of age of treatment (within 27–69 weeks of age), the stage of detachments or mutation (*cmr1/cmr1*, *cmr3/cmr3* or *cmr1/cmr3*). These results show promise for the treatment of human patients with *BEST1* mutations [93].

## 5. Cilia-Related Proteins (Ciliopathies)

Photoreceptors are non-motile sensory ciliated cells. The connecting cilium between inner and outer segment is critical for the transport of proteins to and from the outer segment. Many proteins are expressed in this region of photoreceptors and act to control the trafficking of proteins. Ciliopathies is a general term used to group conditions resulting from mutations in cilia proteins. As cilia are present in many cell types, mutations in cilia proteins can cause syndromic disease where retinal degeneration is present, accompanied by disorders in other organ systems. Mutations that have a milder effect on function may result in a photoreceptor-only phenotype such as LCA or RP. A number of large animal models with mutations in cilia related genes have been identified (Table 1).

### 5.1. BBS4

A mutation in the *Bbs4*, (Bardet–Biedl syndrome 4) gene, was identified in Hungarian Puli dogs with PRA. This mutation (c.58A>T, p.Lys20Ter) is predicted to result in nonsense mediated decay of the *Bbs4* transcript. *BBS4* is one of the BBS genes involved in cilia function. These dogs showed a typical PRA phenotype along with noted obesity and abnormal sperm, although more affected dogs may need to be examined before confirming that this is a syndromic BBS model [95].

### 5.2. BBS7

An NHP model of Bardet–Biedl syndrome has been recently described. A mutation in exon 3 of the *Bbs7* gene c.160delG (p.Ala54GlnfsTer18) that is predicted to produce a truncated non-functional protein was identified. As with BBS4, BBS7 is involved in cilia function. Typically in humans, mutations in BBS genes cause a syndromic condition. The affected NHPs had several features of BBS, including retinal atrophy, which was most severe centrally; the affected animals had smaller brains, renal disease, and the males had small testes. This is the first described model of BBS in NHP and shares many characteristics with BBS patients with truncating mutations [3].

### 5.3. CEP290

This cat model, *rdAc* (retinal degeneration, Abyssinian cat) has been studied in detail for many years. Compared to most *CEP290* (centrosomal protein 290) mutations in other species, the cat model has a mild phenotype. The onset and rate of photoreceptor degeneration in the cat with the sparing of the area centralis makes it a model for RP [96,97]. In humans, *CEP290* mutations can result in a spectrum of phenotypes including lethality, severe syndromic disease (e.g., Joubert syndrome) and most commonly LCA; see [98] for a review. Milder phenotypes such as RP are less common. In the cat, an intronic mutation (c.6966+9T>G) leads to the introduction of a stronger splice acceptor site (the wildtype splice acceptor is a GC rather than the much more commonly used GT). The mutation strengthens an adjacent GT, which, when used, adds 4 bps to the exon 50 sequence, the resulting frameshift introduces a premature stop codon [99]. The truncated protein escapes nonsense mediated decay and is expressed. In addition, the wildtype acceptor site is still used for a percentage of the transcripts (unpublished data). The combination of a truncated protein with some remaining function combined with a low-level production of full-length transcripts explains the mild phenotype. *CEP290* is too large to be packaged in an adeno-associated virus vector and one therapeutic approach being investigated is the use of a truncated transcript so that a miniprotein may have partial function and convert a severe phenotype into a milder phenotype similar to that of the cat.

### 5.4. NPHP4

Wire-haired dachshunds have a cone–rod dystrophy resulting from a truncating mutation in *Nphp4*. The identified *Nphp4* mutation was a deletion involving exon 5/intron 5 that led to skipping of exon 5 and a premature truncation in exon 6 of 30 (c.462_526del, p.Leu155LysfsTer2) [100]. A colony was established from a single founder male [101]. Puppies had miotic pupils and cone-mediated ERGs were reduced prior to retinal maturation. Furthermore, they did not show the normal increase in amplitudes with retinal maturation and further declined in amplitude rapidly. The amplitudes of the rod-driven responses were less severely affected, but were lost with age [102,103]. Interestingly, the condition is non-syndromic in dogs, just presenting as a cone–rod dystrophy. Human patients with *NPHP4* mutations do not always develop a retinal phenotype, but typically have nephronophthisis, with some patients having Senior–Løken syndrome, which combines the renal phenotype with a retinal dystrophy (see for [104] a review). This phenotype difference may represent a species difference, with *Nphp4^−/−^* mice also only having an ocular phenotype with no renal abnormalities, but also showing male infertility [105].

### 5.5. NPHP5 (IQCB1)

A mutation was identified in the *NPHP5* gene (aka *IQCB1*) resulting in a cone–rod dystrophy in American pit bull terrier dogs (*crd2*) modeling non-syndromic LCA. This mutation (c.952-953insC, p.Ser319IlefsTer13) results in a frameshift and a premature stop codon [52]. At 6 weeks of age, the *crd2* dogs had no cone function and abnormal rod function. Morphologically, the cones completely lacked outer segments, while the rods developed disorganized outer segments. Despite the lack of cone function, the cones are retained within the central retina up to 33 weeks of age [106]. Adeno-associated gene therapy restores and improves photoreceptor function and preserves the outer retina layer [107].

### 5.6. RPGR

Retinitis pigmentosa GTPase regulator (RPGR) localizes to the connecting cilium and mutations within the *RPGR* gene account for >80% of X-linked RP (XLRP). There are two major retinal isoforms, one encoded by exons 1–19 and a second isoform that consists of exons 1–15 and a retained portion of intron 15 (ORF15; see [108] for a summary). ORF15 is a mutation hotspot [109]. Three different *Rpgr* mutations cause X-linked retinal degeneration in dogs. One is caused by a genomic deletion [110] and two are due to microdeletions in ORF15 and provide two distinct mechanistic models for RPGR-XLRP [111]. The first, known in the dog as XLPRA1, has a 5 bp deletion and a premature stop codon [111]. This is a relatively late onset and slowly progressive degeneration [112]. The second form, XLPRA2, has a 2 bp deletion with a frameshift resulting in the addition of 34 basic residues. In vitro studies showed that the mutant protein aggregated in the endoplasmic reticulum and is hypothesized to have a toxic effect [111]. The phenotype presents as an early onset degeneration with early changes being outer segment disruption and opsin (rod and cone) mislocalization. Photoreceptor cell death was shown to occur in a biphasic manner with two distinct phases of cell death with evidence of remodeling occurring [113]. Studies of the heterozygous females revealed that there are patches of diseased retina, presumably resulting from regions where random X-inactivation has resulted in expression of the mutant allele and patches of unaffected retina (where the wildtype allele is expressed). In the earlier onset XLPRA2 migration of adjacent photoreceptors into regions where rod photoreceptors died occurs, showing retinal plasticity in the younger animals. This remodeling was not described in the XLPRA1, where patches of degeneration occur at a later age [114].

### 5.7. RPGRIP1

Retinitis pigmentosa GTPase regulator-interacting protein 1 (RPGRIP1) localizes to the connecting cilium, where it interacts with RPGR. Mutations in *RPGRIP1* are associated with autosomal recessive LCA. A cone–rod dystrophy form of PRA in a colony of miniature longhaired Dachshunds was reported to be due to an insertion in *Rpgrip1* [115] and the rescue of the phenotype by gene therapy was achieved [116]. When miniature longhaired Dachshunds in pet homes were investigated, the *Rpgrip1* insertion did not appear to segregate with disease status [117]. Further studies have shown that two other loci influence the development of the phenotype [118,119,120]. This is an example of the potential effect of modifying loci on phenotype.

## 6. Photoreceptor Development

### 6.1. CRX

The *CRX* gene encodes an OTX-like homeodomain transcription factor which is essential for photoreceptor development, maturation and survival [121,122,123,124]. Transcription factors are essential for control of the maturation of progenitor cells [123,124,125,126]. Only one spontaneously occurring large animal model of a retinal transcription factor homeobox mutation has been described, the *Crx^Rdy^* cat [127,128]. The heterozygous animal has a severe phenotype of cone-led retinal dystrophy and is a model for one form of severe early childhood onset blindness (LCA7). While most mutations in *CRX* result in LCA7, other phenotypes including cone–rod dystrophy, RP and macular degeneration have been described [129,130,131]. *CRX* mutations have been reported to account for between 0.6% and 2.35% of LCA [131,132,133,134].

As with other transcription factors, CRX has a characteristic structure with a DNA binding domain (homeodomain) near its N-terminal and a transactivation domain at the C-terminal. The *Crx^Rdy/+^* mutant cat models Class III *CRX* mutations, which are antimorphic frameshift/nonsense mutations with intact DNA binding, but a lack of target gene transactivation [135]. The *Crx^Rdy/+^* cat mutation is caused by a 1 bp deletion (c.546delC; p.Pro185LysfsTer2) in the final exon of the *Crx* gene [127]. This mutant mRNA avoids nonsense mediated decay and produces a truncated Crx protein with an intact DNA-binding domain but disrupted transactivation domain [128]. Both the mutant transcript and protein accumulate at higher levels than the wildtype versions, possibly due to increased stability of the mutant mRNA compared to the wildtype. In vitro studies showed that the mutant mRNA fails to activate its own promoter [128]. This suggests that the mutant protein has a dominant negative effect by binding promoter recognition sites, but fails in its transactivation function. The result is misregulated gene expression; for example, *Rho* and cone arrestin (*Arr3*) mRNA levels are severely decreased. Truncated photoreceptor outer segments are produced, but the photoreceptors fail to fully mature [128]. A decreased rod ERG is detectable, which shows evidence of partial maturation before this is halted and the ERG amplitudes decline, paralleling rod degeneration. Cone function is more severely affected, with no cone ERG responses being detectable [128].

### 6.2. STK38L

An early retinal degeneration was described in Norwegian Elkhounds in 1987 [136] in which the dogs present with vision impairment in low light as early as 6 weeks of age. The causal mutation was found to be a short interspersed element (SINE) insertion in exon 4 of *Stk38l*, a gene that had not been associated with retinal degenerations before its discovery in the Norwegian Elkhounds [137]. The mRNA transcript is produced in the affected dog but does not contain exon 4. Interestingly, at ~6 weeks of age, the expression of the mutant *Stk38l* transcript is similar to control dogs, but expression increases at ~8 and ~12 weeks [138].

Detailed histological experiments were performed on affected canine retina to understand the role of Stk38l in retinal development and how the lack of the functional protein results in retinal degeneration. The retina in ~4 week old *Stk38l*-affected dogs is normal, but by ~8 weeks of age the rod photoreceptors begin to show mislocalization of Rho, irregularities in the outer segments and increased TUNEL labeling. However, this increase in apoptotic cells is not accompanied by a thinning of the outer nuclear layer (ONL), as would be expected from loss of rod photoreceptors. The maintenance of ONL thickness is a result of proliferation of rod-like photoreceptors which express both Rho and cone S-opsin. After ~14 weeks of age, this proliferation ceases and the ONL begins to thin as the photoreceptors die, resulting in the loss of at least 50% of the ONL rows by 48 weeks [138].

The *Stk38l*-affected dogs provide an interesting and unique model of retinal degeneration in which differentiated photoreceptors either apoptose or proliferate for a short amount of time before retinal degeneration progresses.

## 7. Photoreceptor to Bipolar Cell Signaling

### 7.1. LRIT3

Recently, a dog model of recessively inherited CSNB has been identified due to a mutation in leucine-rich-repeat, immunoglobulin-like transmembrane-domain 3 gene (*LRIT3*). Mutations in *LRIT3* in humans causes a form of CSNB [139]. The ERG of the affected dogs shows a lack of ON-bipolar cell function with preservation of cone OFF-bipolar contributions [140,141,142]. There is currently debate about the positioning of the LRIT3 protein which had been described as being in the synaptic tips of the bipolar cells; however, a recent publication showed that it was presynaptic, being present in photoreceptors, but bridged the synapse to influence the positioning of post-synaptic glutamate signaling proteins [143]. The new availability of a large animal model may facilitate further investigations.

### 7.2. TRPM1

Studies of Appaloosa horses with CSNB contributed to the identification of the role of transient receptor potential cation channel subfamily M, member 1 (*Trpm1*) in bipolar cell signaling [144,145]. The night-blind Appaloosa horse was first recognized as an animal model for the Schubert–Bornschein type of CSNB in the 1970s [146]. The study published at that time reported a lack of an ERG b-wave and night blindness but no retinal degeneration or obvious morphological abnormality of the photoreceptor synapses with second order neurons [146]. The correlation of CSNB in the Appaloosa with the Leopard complex spotting coat color was recognized [147]. This coat color is governed at a single gene locus with animals homozygous for the Leopard complex spotting associated allele (LP) also having CSNB [147]. When the LP locus was mapped, *Trpm1*, which is also expressed in melanocytes, was identified as a positional candidate gene and showed markedly reduced expression in retina and skin from affected animals [148]. Investigation of *TRPM1* in humans with complete CSNB identified mutations in *TRPM1* [149,150,151] and its role in ON-bipolar cell signaling was identified.

The LP mutation was identified as a retroviral insertion in intron 1 of equine *Trpm1* that disrupts gene transcription by causing premature polyadenylation [152].

### 7.3. Whippet Dog Model of Incomplete CSNB with Retinal Degeneration

A dog model of cone–rod synaptic dysfunction, which has been described by some authors as a form of incomplete CSNB, has been identified [153]. The affected dogs lack an ERG b-wave and also lack cone OFF-bipolar cell attributable ERG components [154]. Interestingly, the dog model develops a progressive retinal degeneration, which is not reported as a feature of the condition in humans [155].

## 8. Structural/Other

Retinal degenerations can occur due to mutations in genes involved in the structure or maintenance of the retina.

### 8.1. ADAM9

ADAM9 (a disintegrin and metalloprotease domain, family member 9) mutations are associated with cone–rod dystrophies in humans and dogs. A genomic deletion in *Adam9* was identified in Irish Glen of Imaal Terriers, resulting in a premature stop codon and the loss of the full-length protein. Similar to the mouse model, histological sections show that the RPE cells do not invest in the outer segments of the photoreceptors. This mutation effects both the rod and cone photoreceptors, but the cones are more severely affected throughout the course of the disease [156,157]. Further work is needed to understand the role of ADAM9 in the retinal structure and RPE maintenance of photoreceptors.

### 8.2. AIPL1

Mutations in the gene encoding aryl hydrocarbon receptor-interacting protein-like 1 (*AIPL1*) causes LCA4 accounting for about 3% to 7% of autosomal recessive LCA [132,134,158,159,160]. The *AIPL1* gene encodes for a protein expressed in the photoreceptor and pineal gland [161]. In photoreceptors, AIPL1 is concentrated in the tip of the inner segment in proximity to the connecting cilia, acting as a co-chaperone involved in the folding, assembly and transport of the retinal cGMP phosphodiesterase (PDE6) heteromeric holoenzyme within photoreceptor outer segments [162,163,164,165,166].

One spontaneous occurring feline large animal model of LCA4 currently exists, the Persian cat [167]. This feline model, similar to human patients with LCA4, presents as an autosomal recessive severe retinal dystrophy. The disease is characterized by a very early photoreceptor loss, progressing to severe retinal degeneration before adulthood. This leads to very early blindness [167]. The feline phenotype is caused by a nonsense mutation at position c.577C>T producing a stop codon (p.Arg193Ter), leading to the production of a truncated non-functional protein [168]. The precise mechanisms of the severe phenotype in the feline model are yet to be investigated. The feline model represents a valuable large animal model for mechanistic studies underlying *AIPL1*-LCA in humans.

### 8.3. MERTK

Mutations in *MERTK* (MER proto-concogene, tyrosine kinase) account for about 1% of arRP. *Mertk* was found to be overexpressed (six-fold) in Swedish Vallhund dogs with a retinal dystrophy [169]. After whole genome sequencing of an affected dog, an intact LINE-1 insertion was identified in intron one of *Mertk*. A LINE-1 retrotransposon is a long interspersed repetitive element that is found in all mammalian genomes [170]. It is unclear how this insertion results in overexpression of the *Mertk* transcript and subsequent development of retinal disease. More in-depth investigation of this mechanism will aid a better understanding of how retrotransposon insertions can modify gene regulation/expression, ultimately leading to disease, in addition to understanding the specific role of overexpression of the *MERTK* transcript in the context of retinal disease [171].

### 8.4. PRCD

Progressive rod–cone degeneration (*Prcd*) was a formerly unknown gene that was mapped and sequenced first in dogs with progressive rod–cone degeneration (PRCD) PRA [172]. Dogs with this form of PRA had been studied in detail over many years and many different breeds of dog are affected. The mutation is p.Cys2Tyr, which is in a highly conserved region of the gene. An identical mutation was found in a human RP patient. Additional mutations also located in exon 1 of *PRCD* have been identified in RP patients [172,173]. Since the discovery of *Prcd*, a mouse knockout model has been generated and experiments have shown Prcd’s involvement in photoreceptor disc formation and maintenance [174,175,176]. Studies in the PRCD dog showed an early ultrastructural change that may have resulted from this purported function. This was the development of vesicular profiles adjacent to outer segments being consistent with abnormality in disc formation, furthermore studies also showed affected dogs had slower photoreceptor disc renewal than normal controls [177].

### 8.5. RD3

Mutations in *RD3* result in LCA12 in humans, a severe retinal degeneration in mice and rod–cone dysplasia type 2 (*rcd2*) in Collie dogs [178,179,180]. RD3 is thought to act as competitor for guanylyl cyclase-activating proteins, preventing premature cyclase activity in inner segments [181].

Collie dogs have a 22 bp insertion in the *Rd3* gene resulting in altered amino acids and an extended open reading frame [182]. Three splice variants of the gene were identified in the canine retina. In the *Rd3*-mutant Collie, the rod and cone photoreceptors outer segments do not fully develop and the retina degenerates rapidly. By 6 months, there are no photoreceptor inner or outer segments and the outer nuclear layer contains only one layer of nuclei [183].

## 9. Conclusions

IRDs that lead to visual impairment and blindness show considerable heterogeneity, where even similar disease phenotypes can result from mutations in a wide range of different genes. Closer examination of the broad phenotypes such as RP and LCA show the genetic heterogeneity with altered function of a range of genes leading to photoreceptor loss. Large numbers of different mutations within a single gene exist and can result in a range of phenotypic differences.

The mechanisms by which the gene mutation leads to cell death differs between genes and between mutations within individual genes. Laboratory rodents have been the workhorses for studying disease mechanisms because of the ease with which their genome can be engineered, the short reproductive time and relatively low costs of maintenance. They have allowed identification of disease pathways and allowed the study of how and why degeneration occurs. However, laboratory rodents have very significant shortcomings. Rodents are nocturnal animals with a different pattern of distribution of photoreceptors to humans. The large animal models discussed in this review have retinal photoreceptor distribution that is much closer to that of humans. This is particularly important when considering conditions that have a major effect on the macula. Large animal models have also been of value for the development and testing of translational therapies. They are particularly valuable for this because the larger eye size and proportions of lens and vitreous is much closer to that in humans, allowing for identical surgical approaches for trials of translational therapies. The several, mostly spontaneously occurring, large animal models mentioned here have provided valuable information about disease phenotypes and disease mechanisms. They offer great potential for even more detailed studies to understand how photoreceptor function and survivability is affected by the gene mutations and to study the extensive inner retinal remodeling and glial cell activation that occurs in IRDs. Modeling of IRDs using retinal organoids developed from induced pluripotent stem cells from IRD patients is an exciting field of research. These potentially allow the study of the effect of the exact mutation that is present in the patient and in a human cultured tissue. However, they do not yet fully recapitulate the specific environment within the retina of a living animal, so, while showing great promise, they cannot yet replace whole animal studies. Improving our understanding of how and why photoreceptors die may suggest novel therapies to preserve function and slow down vision loss. There remain untapped populations of companion animals (dogs and cats) with spontaneous IRDs, with new potential models being identified with increasing frequency. The advances in gene editing also expand the opportunity to develop additional large animal models with specific mutations that even more accurately model human IRDs.

## Figures and Tables

**Table 1:** List of mutations in large animal models of inherited retinal disease. Detailed information for some genes (indicated with*) are displayed in the text, highlighting the role of large animal models in advancing the understanding of the disease mechanism and/or providing insights into normal protein structure and function. References are available in Supplementary Material Table S1.

Mechanism	Gene	Species	Mutation
Phototransduction	RHO*	dog pig pig pig	c.11C>G, p.Thr4Arg p.Pro23His c.1040C>T, p.Pro347Leu p.Pro347Ser
	PDE6A*	dog	c.1939delA, p.Asn616ThrfsTer39
	PDE6B*	dog	c.2420G>A, p.Trp807Ter; c.2449_2450insTGAAGTCC; p.Lys816Terfs817 c.2404_2406delAAC, p.Asn802del
	PDE6C*	NHP	c.1694G>A, p.Arg565Gln
	*SAG*	dog	c.1216T>C; p.Ter406extArg*25
Visual Cycle	ABCA4*	dog	c.4176insC, p.Phe1393LeufsTer3
	RPE65*	dog	c.487_490delAAGA; p.Lys154LeufsTer53
	RDH5*	cat	unpublished
Channelopathies/channel related	CNGA1*	dog	c.1752_1755delAACT, p.Thr584SerfsTer9
	CNGB1*	dog	c.2387delA;2389_2390insAGCTAC, p.Ser791ArgfsTer2
	CNGA3*	dog dog sheep sheep	c.1270C>T; p.Arg424Trp c.1931_1933delTGG, p.Val644del p.Arg236Ter p.Gly540Ser
	CNGB3*	dog	c.784G>A; p.Asp262Asn CFA29:g.35,699,378-36,104,197del, c.0
	BEST1*	dog	c.73C>T, p.Arg25Ter; c.482G>A, p.Gly161Asp; c.C1388del and c.1466G>T, p.Pro463fs and p.Gly489Val
Ciliopathies	BBS4*	dog	c.58A>T, p.Lys20Ter
	BBS7*	NHP	c.160delG, p.Ala54GlnfsTer18
	*c2orf71*	dog	c.3149_3150insC, p.Lys1051ValfsTer91
	*CCDC66*	dog	c.521_522insA, p.Asn174LysfsTer2
	CEP290*	cat	c.6966+9T>G, p.Ile2323AlafsTer3
	*FAM161A*	dog	c.1758-15_1758-16ins238, p.Ser588MetfsTer14
	NPHP4*	dog	c.462_526del, p.Leu155LysfsTer2
	NPHP5(IQCB1)*	dog cat	c.952-953insC, p.Ser319IlefsTer13 c.1282delCT, p.Leu428Ter
	RPGR*	dog	c.1084-1085delGA, c.1028-1032delGAGAA CFAX:g. 33106747+190-33102324del
	RGRIP1*	dog	CFA15:g.8228_8229insA29GGAAGCAACAGGATG
	*TTC8*	dog	c.669delA, p.Lys223ArgfsTer15
Photoreceptor development	CRX*	cat	c.546delC, p.Pro185LysfsTer2
	STK38L*	dog	c.299_300ins [218;285_299]; p.Lys63_Glu103del
Photoreceptor to Bipolar Cell	LRIT3*	dog	c.762_763delG, p.Lys246AsnfsTer5
	TRPM1*	horse	ECA1g.108,297,929_108,297,930ins1378
	Whippet*	dog	unpublished
Structural/Other	ADAM9*	dog	c.1592_1881del p.Lys531AsnfsTer3
	AIPL1*	cat	c.577C>T, p.Arg193Ter
	MERTK*	dog	CFA17:g.36338057_36338058ins[(6401);36338043-36338057]
	PRCD*	dog	c.5G>A, p.Cys2tyr
	RD3*	dog	c.418_419ins[22]
	*NECAP1*	dog	c.544G>A, p.Gly182Arg
	*PPT1*	dog	CFA15:g.[2,866,454_2,877,574dup; 2,874,661_2,875,048con2,877,563-2,877,607inv]
	*SLC4A3*	dog	c.2601_2602insC, p.Glu868ArgfsTer104

## Supplementary Materials

The following are available online at https://www.mdpi.com/2073-4409/9/4/882/s1, Table S1: List of mutations in large animal models of inherited retinal disease and references.
