# Supplementary material for: Large Animal Models of Inherited Retinal Degenerations: A Review
Source: Cells. 2020 Apr 3;9(4):882. doi: 10.3390/cells9040882 (PMC7226744; doi:10.3390/cells9040882)
Supplement: Supplementary file 1 [file cells-09-00882-s001.pdf]

Supplemental Table 1. List of mutations in large animal models of inherited retinal disease and references.

**Mechanism**	**Gene**	**Species**	**Mutation**	**Reference**
Phototransduction	*RHO*	dog<br>pig<br>pig<br>pig	c. 11C>G, p.Thr4Arg<br>p.Pro23His<br>c.1040C>T, p.Pro347Leu<br>p.Pro347Ser	[1]<br>[2]<br>[3]<br>[4]
	PDE6A	dog	c.1939delA, p.Asn616ThrfsTer39	[5,6]
	PDE6B	dog	c.2420G>A, p.Trp807Ter;<br>c.2449_2450insTGAAGTCC; p.Lys816Terfs817<br>c.2404_2406delAAC, p.Asn802del	[7]<br>[8]<br>[9]
	PDE6C	NHP	c.1694G>A, p.Arg565Gln	[10]
	SAG	dog	c.1216T>C; p.Ter406extArg25	[11]
Visual Cycle	*ABCA4*	dog	c.4176insC, p.Phe1393LeufsTer3	[12]
	RPE65	dog	c.487_490delAAGA; p.Lys154LeufsTer53	[13]
	RDH5	cat	unpublished	
Channelopathies/channel related	*CNGA1*	dog	c.1752_1755delAACT, p.Thr584SerfsTer9	[14]
	CNGB1	dog	c.2387delA;2389_2390insAGCTAC, p.Ser791ArgfsTer2	[15]
	CNGA3	dog<br>dog<br>sheep<br>sheep	c.1270C>T; p.Arg424Trp<br>c.1931_1933delTGG, p.Val644del<br>p.Arg236Ter<br>p.Gly540Ser	[16]<br><br>[17]<br>[18]
	CNGB3	dog	c.784G>A; p.Asp262Asn<br>CFA29:g.35,699,378-36,104,197del, c.0	[19]<br>[20]
	BEST1	dog	c.73C>T, p.Arg25Ter;<br>c.482G>A, p.Gly161Asp;<br>c.C1388del and c.1466G>T, p.Pro463fs and p.Gly489Val	[21]<br><br>[22]

Ciliopathies	*BBS4*	dog	c.58A->T, p.Lys20Ter	[23]
	BBS7	NHP	c.160delG, p.Ala54GlnfsTer18	[24]
	c2orf71	dog	c.3149_3150insC, p.Lys1051ValfsTer91	[25]
	CCDC66	dog	c.521_522insA, p.Asn174LysfsTer2	[26]
	CEP290	cat	c.6966+9T>G, p.Ile2323AlafsTer3	[27]
	FAM161A	dog	c.1758-15_1758-16ins238, p.Ser588MetfsTer14	[28]
	NPHP4	dog	c.462_526del, p.Leu155LysfsTer2	[29]
	NPHP5(IQCB1)	dog<br>cat	c.952-953insC, p.Ser319IlefsTer13<br>c.1282delCT, p.Leu428Ter	[9]<br>[30]
	RPGR	dog	c.1084-1085delGA,<br>c.1028-1032delGAGAA<br>CFAX:g. 33106747+190-33102324del	[31]<br>[32]
	RGRIP1	dog	CFA15:g.8228_8229insA29GGAAGCAACAG GATG	[33]
	TTC8	dog	c.669delA, p.Lys223ArgfsTer15	[34]
Photoreceptor development	*CRX*	cat	c.546delC, p.Pro185LysfsTer2	[35]
	STK38L	dog	c.299_300ins[218;285_299]; p.Lys63_Glu103del	[36]
Photoreceptor to Bipolar Cell	*LRIT3*	dog	c.762_763delG, p.Lys246AsnfsTer5	[37]
	TRPM1	horse	ECA1g.108,297,929_108,297,930 ins1378	[38]
	Whippet	dog	unpublished	
Structural/Other	*ADAM9*	dog	c.1592_1881del p.Lys531AsnfsTer3	[39,40]
	AIPL1	cat	c.577C>T, p.Arg193Ter	[41]
	MERTK	dog	CFA17:g.36338057_36338058ins[(6401);36338043-36338057]	[42]

	PRCD	dog	c.5G>A, p.~~C2Y~~Cys2Tyr	[43]
	RD3	dog	c.418_419ins[22]~~, p.Pro139AlafsTer69~~	[44,45]
	NECAP1	dog	c.544G>A, p.Gly182Arg	[46]
	PPT1	dog	CFA15:g.[2,866,454_2,877,574dup;<br>2,874,661_2,875,048con2,877,563-2,877,607inv]	[47]
	SLC4A3	dog	c.2601_2602insC, p.Glu868ArgfsTer104	[48]

1. Kijas, J.W.; Cideciyan, A.V.; Aleman, T.S.; Pianta, M.J.; Pearce-Kelling, S.E.; Miller, B.J.; Jacobson, S.G.; Aguirre, G.D.; Acland, G.M. Naturally occurring rhodopsin mutation in the dog causes retinal dysfunction and degeneration mimicking human dominant retinitis pigmentosa. *Proc Natl Acad Sci U S A* **2002**, *99*, 6328-6333, doi:10.1073/pnas.082714499 082714499 [pii].
2. Ross, J.W.; Fernandez de Castro, J.P.; Zhao, J.; Samuel, M.; Walters, E.; Rios, C.; Bray-Ward, P.; Jones, B.W.; Marc, R.E.; Wang, W., et al. Generation of an inbred miniature pig model of retinitis pigmentosa. *Invest Ophthalmol Vis Sci* **2012**, *53*, 501-507, doi:10.1167/iovs.11-8784.
3. Petters, R.M.; Alexander, C.A.; Wells, K.D.; Collins, E.B.; Sommer, J.R.; Blanton, M.R.; Rojas, G.; Hao, Y.; Flowers, W.L.; Banin, E., et al. Genetically engineered large animal model for studying cone photoreceptor survival and degeneration in retinitis pigmentosa. *Nat Biotechnol* **1997**, *15*, 965-970.
4. Kraft, T.W.; Allen, D.; Petters, R.M.; Hao, Y.; Peng, Y.W.; Wong, F. Altered light responses of single rod photoreceptors in transgenic pigs expressing P347L or P347S rhodopsin. *Mol Vis* **2005**, *11*, 1246-1256.
5. Petersen-Jones, S.M.; Entz, D.D.; Sargan, D.R. cGMP phosphodiesterase-α mutation causes progressive retinal atrophy in the Cardigan Welsh corgi dog. *Invest Ophthalmol Vis Sci* **1999**, *40*, 1637-1644.
6. Tuntivanich, N.; Pittler, S.J.; Fischer, A.J.; Omar, G.; Kiupel, M.; Weber, A.; Yao, S.; Steibel, J.P.; Khan, N.W.; Petersen-Jones, S.M. Characterization of a Canine Model of Autosomal Recessive Retinitis Pigmentosa due to a PDE6A Mutation. *Inv Ophthalmol and Vis Sci* **2009**, *50*, 801-813, doi:10.1167/iovs.08-2562.
7. Suber, M.L.; Pittler, S.J.; Quin, N.; Wright, G.C.; Holcombe, N.; Lee, R.H.; Craft, C.M.; Lolley, R.N.; Baehr, W.; Hurwitz, R.L. Irish setter dogs affected with rod-cone dysplasia contain a nonsense mutation in the rod cGMP phosphodiesterase beta-subunit gene. *Proc Natl Acad Sci U S A* **1993**, *90*, 3968-3972.
8. Dekomien, G.; Runte, M.; Godde, R.; Epplen, J.T. Generalized progressive retinal atrophy of Sloughi dogs is due to an 8-bp insertion in exon 21 of the PDE6B gene. *Cytogenet Cell Genet* **2000**, *90*, 261-267.

9. Goldstein, O.; Mezey, J.G.; Schweitzer, P.A.; Boyko, A.R.; Gao, C.; Bustamante, C.D.; Jordan, J.A.; Aguirre, G.D.; Acland, G.M. IQCB1 and PDE6B mutations cause similar early onset retinal degenerations in two closely related terrier dog breeds. *Invest Ophthalmol Vis Sci* **2013**, *54*, 7005-7019, doi:10.1167/iovs.13-12915.
10. Moshiri, A.; Chen, R.; Kim, S.; Harris, R.A.; Li, Y.; Raveendran, M.; Davis, S.; Liang, Q.; Pomerantz, O.; Wang, J., et al. A nonhuman primate model of inherited retinal disease. *J Clin Inv* **2019**, *129*, 863-874, doi:10.1172/JCI123980.
11. Goldstein, O.; Jordan, J.A.; Aguirre, G.D.; Acland, G.M. A non-stop S-antigen gene mutation is associated with late onset hereditary retinal degeneration in dogs. *Mol Vis* **2013**, *19*, 1871-1884.
12. Makelainen, S.; Godia, M.; Hellsand, M.; Viluma, A.; Hahn, D.; Makdoumi, K.; Zeiss, C.J.; Mellersh, C.; Ricketts, S.L.; Narfstrom, K., et al. An ABCA4 loss-of-function mutation causes a canine form of Stargardt disease. *PLoS Genet* **2019**, *15*, e1007873, doi:10.1371/journal.pgen.1007873.
13. Aguirre, G.D.; Baldwin, V.; Pearce-Kelling, S.; Narfström, K.; Ray, K.; Acland, G.M. Congenital stationary night blindness in the dog: common mutation in the RPE65 gene indicates founder effect. *Mol Vis* **1998**, *4*, 23.
14. Wiik, A.C.; Ropstad, E.O.; Ekesten, B.; Karlstam, L.; Wade, C.M.; Lingaas, F. Progressive retinal atrophy in Shetland sheepdog is associated with a mutation in the CNGA1 gene. *Anim Genet* **2015**, *46*, 515-521, doi:10.1111/age.12323.
15. Winkler, P.A.; Ekenstedt, K.J.; Occelli, L.M.; Frattaroli, A.V.; Bartoe, J.T.; Venta, P.J.; Petersen-Jones, S.M. A large animal model for *CNGB1* autosomal recessive retinitis pigmentosa. *PLoS One* **2013**, *8*, e72229, doi:10.1371/journal.pone.0072229.
16. Tanaka, N.; Dutrow, E.V.; Miyadera, K.; Delemotte, L.; MacDermaid, C.M.; Reinstein, S.L.; Crumley, W.R.; Dixon, C.J.; Casal, M.L.; Klein, M.L., et al. Canine CNGA3 Gene Mutations Provide Novel Insights into Human Achromatopsia-Associated Channelopathies and Treatment. *PLoS One* **2015**, *10*, e0138943, doi:10.1371/journal.pone.0138943.
17. Reicher, S.; Seroussi, E.; Gootwine, E. A mutation in gene CNGA3 is associated with day blindness in sheep. *Genomics* **2010**, *95*, 101-104, doi:10.1016/j.ygeno.2009.10.003.
18. Gootwine, E.; Abu-Siam, M.; Obolensky, A.; Rosov, A.; Honig, H.; Nitzan, T.; Shirak, A.; Ezra-Elia, R.; Yamin, E.; Banin, E., et al. Gene Augmentation Therapy for a Missense Substitution in the cGMP-Binding Domain of Ovine CNGA3 Gene Restores Vision in Day-Blind Sheep. *Invest Ophthalmol Vis Sci* **2017**, *58*, 1577-1584, doi:10.1167/iovs.16-20986.
19. Sidjanin, D.J.; Lowe, J.K.; McElwee, J.L.; Milne, B.S.; Phippen, T.M.; Sargan, D.R.; Aguirre, G.D.; Acland, G.M.; Ostrander, E.A. Canine CNGB3 mutations establish cone degeneration as orthologous to the human achromatopsia locus ACHM3. *Hum Mol Gen* **2002**, *11*, 1823-1833.
20. Yeh, C.Y.; Goldstein, O.; Kukekova, A.V.; Holley, D.; Knollinger, A.M.; Huson, H.J.; Pearce-Kelling, S.E.; Acland, G.M.; Komaromy, A.M. Genomic deletion of CNGB3 is identical by descent in multiple canine breeds and causes achromatopsia. *BMC Genet* **2013**, *14*, 27, doi:10.1186/1471-2156-14-27.
21. Guziewicz, K.E.; Zangerl, B.; Lindauer, S.J.; Mullins, R.F.; Sandmeyer, L.S.; Grahn, B.H.; Stone, E.M.; Acland, G.M.; Aguirre, G.D. Bestrophin gene mutations cause canine

multifocal retinopathy: a novel animal model for best disease. *Invest Ophthalmol Vis Sci* **2007**, *48*, 1959-1967, doi:48/5/1959 [pii]
10.1167/iovs.06-1374.

22. Zangerl, B.; Wickstrom, K.; Slavik, J.; Lindauer, S.J.; Ahonen, S.; Schelling, C.; Lohi, H.; Guziewicz, K.E.; Aguirre, G.D. Assessment of canine BEST1 variations identifies new mutations and establishes an independent bestrophinopathy model (cmr3). *Mol Vis* **2010**, *16*, 2791-2804, doi:299 [pii].
23. Chew, T.; Haase, B.; Bathgate, R.; Willet, C.E.; Kaukonen, M.K.; Mascord, L.J.; Lohi, H.T.; Wade, C.M. A Coding Variant in the Gene Bardet-Biedl Syndrome 4 (BBS4) Is Associated with a Novel Form of Canine Progressive Retinal Atrophy. *G3-Genes Genom Genet* **2017**, *7*, 2327-2335, doi:10.1534/g3.117.043109.
24. Peterson, S.M.; McGill, T.J.; Puthussery, T.; Stoddard, J.; Renner, L.; Lewis, A.D.; Colgin, L.M.A.; Gayet, J.; Wang, X.; Prongay, K., et al. Bardet-Biedl Syndrome in rhesus macaques: A nonhuman primate model of retinitis pigmentosa. *Exp Eye Res* **2019**, *189*, 107825, doi:10.1016/j.exer.2019.107825.
25. Downs, L.M.; Bell, J.S.; Freeman, J.; Hartley, C.; Hayward, L.J.; Mellersh, C.S. Late-onset progressive retinal atrophy in the Gordon and Irish Setter breeds is associated with a frameshift mutation in C2orf71. *Anim Genet* **2013**, *44*, 169-177, doi:10.1111/j.1365-2052.2012.02379.x.
26. Dekomien, G.; Vollrath, C.; Petrasch-Parwez, E.; Boeve, M.H.; Akkad, D.A.; Gerding, W.M.; Epplen, J.T. Progressive retinal atrophy in Schapendoes dogs: mutation of the newly identified CCDC66 gene. *Neurogenetics* **2010**, *11*, 163-174, doi:10.1007/s10048-009-0223-z.
27. Menotti-Raymond, M.; David, V.A.; Schaffer, A.A.; Stephens, R.; Wells, D.; Kumar-Singh, R.; O'Brien, S.J.; Narfström, K. Mutation in CEP290 discovered for cat model of human retinal degeneration. *J Hered* **2007**, *98*, 211-220.
28. Downs, L.M.; Mellersh, C.S. An Intronic SINE insertion in FAM161A that causes exon-skipping is associated with progressive retinal atrophy in Tibetan Spaniels and Tibetan Terriers. *PLoS One* **2014**, *9*, e93990, doi:10.1371/journal.pone.0093990.
29. Wiik, A.C.; Wade, C.; Biagi, T.; Ropstad, E.O.; Bjerkas, E.; Lindblad-Toh, K.; Lingaas, F. A deletion in nephronophthisis 4 (NPHP4) is associated with recessive cone-rod dystrophy in standard wire-haired dachshund. *Genome Res* **2008**, *18*, 1415-1421.
30. Oh, A.; Pearce, J.W.; Gandolfi, B.; Creighton, E.K.; Suedmeyer, W.K.; Selig, M.; Bosiack, A.P.; Castaner, L.J.; Whiting, R.E.; Belknap, E.B., et al. Early-Onset Progressive Retinal Atrophy Associated with an IQCB1 Variant in African Black-Footed Cats (Felis nigripes). **2017**.
31. Zhang, Q.; Acland, G.M.; Wu, W.X.; Johnson, J.L.; Pearce-Kelling, S.; Tulloch, B.; Vervoort, R.; Wright, A.F.; Aguirre, G.D. Different RPGR exon ORF15 mutations in Canids provide insights into photoreceptor cell degeneration. *Hum Mol Gen* **2002**, *11*, 993-1003.
32. Kropatsch, R.; Akkad, D.A.; Frank, M.; Rosenhagen, C.; Altmuller, J.; Nurnberg, P.; Epplen, J.T.; Dekomien, G. A large deletion in RPGR causes XLPRA in Weimaraner dogs. *Canine Genet Epidemiol* **2016**, *3*, 7, doi:10.1186/s40575-016-0037-x.
33. Mellersh, C.S.; Boursnell, M.E.; Pettitt, L.; Ryder, E.J.; Holmes, N.G.; Grafham, D.; Forman, O.P.; Sampson, J.; Barnett, K.C.; Blanton, S., et al. Canine RPGRIP1 mutation establishes cone-rod dystrophy in miniature longhaired dachshunds as a homologue of

human Leber congenital amaurosis. *Genomics* **2006**, *88*, 293-301, doi:S0888-7543(06)00148-0 [pii]
10.1016/j.ygeno.2006.05.004.
34. Downs, L.M.; Wallin-Hakansson, B.; Bergstrom, T.; Mellersh, C.S. A novel mutation in TTC8 is associated with progressive retinal atrophy in the golden retriever. *Canine Genet Epidemiol* **2014**, *1*, 4, doi:10.1186/2052-6687-1-4.
35. Menotti-Raymond, M.; Deckman, K.H.; David, V.; Myrkalo, J.; O'Brien, S.J.; Narfstrom, K. Mutation discovered in a feline model of human congenital retinal blinding disease. *Invest Ophthalmol Vis Sci* **2010**, *51*, 2852-2859, doi:10.1167/iovs.09-4261.
36. Goldstein, O.; Kukekova, A.V.; Aguirre, G.D.; Acland, G.M. Exonic SINE insertion in STK38L causes canine early retinal degeneration (erd). *Genomics* **2010**, *96*, 362-368, doi:S0888-7543(10)00198-9 [pii]
10.1016/j.ygeno.2010.09.003.
37. Zeitz, C.; Jacobson, S.G.; Hamel, C.P.; Bujakowska, K.; Neuille, M.; Orhan, E.; Zanlonghi, X.; Lancelot, M.E.; Michiels, C.; Schwartz, S.B., et al. Whole-exome sequencing identifies LRIT3 mutations as a cause of autosomal-recessive complete congenital stationary night blindness. *Am J Hum Genet* **2013**, *92*, 67-75, doi:S0002-9297(12)00584-8 [pii]
10.1016/j.ajhg.2012.10.023.
38. Bellone, R.R.; Holl, H.; Setaluri, V.; Devi, S.; Maddodi, N.; Archer, S.; Sandmeyer, L.; Ludwig, A.; Foerster, D.; Pruvost, M., et al. Evidence for a retroviral insertion in TRPM1 as the cause of congenital stationary night blindness and leopard complex spotting in the horse. *PLoS One* **2013**, *8*, e78280, doi:10.1371/journal.pone.0078280.
39. Kropatsch, R.; Petrasch-Parwez, E.; Seelow, D.; Schlichting, A.; Gerding, W.M.; Akkad, D.A.; Epplen, J.T.; Dekomien, G. Generalized progressive retinal atrophy in the Irish Glen of Imaal Terrier is associated with a deletion in the ADAM9 gene. *Mol Cell Probe* **2010**, *24*, 357-363, doi:S0890-8508(10)00047-2 [pii]
10.1016/j.mcp.2010.07.007.
40. Goldstein, O.; Mezey, J.G.; Boyko, A.R.; Gao, C.; Wang, W.; Bustamante, C.D.; Anguish, L.J.; Jordan, J.A.; Pearce-Kelling, S.E.; Aguirre, G.D., et al. An ADAM9 mutation in canine cone-rod dystrophy 3 establishes homology with human cone-rod dystrophy 9. *Mol Vis* **2010**, *16*, 1549-1569, doi:167 [pii].
41. Lyons, L.A.; Creighton, E.K.; Alhaddad, H.; Beale, H.C.; Grahn, R.A.; Rah, H.; Maggs, D.J.; Helps, C.R.; Gandolfi, B. Whole genome sequencing in cats, identifies new models for blindness in AIPL1 and somite segmentation in HES7. *BMC Genomics* **2016**, *17*, 265, doi:10.1186/s12864-016-2595-4.
42. Everson, R.; Pettitt, L.; Forman, O.P.; Dower-Tylee, O.; McLaughlin, B.; Ahonen, S.; Kaukonen, M.; Komaromy, A.M.; Lohi, H.; Mellersh, C.S., et al. An intronic LINE-1 insertion in MERTK is strongly associated with retinopathy in Swedish Vallhund dogs. *PLoS One* **2017**, *12*, e0183021, doi:10.1371/journal.pone.0183021.
43. Goldstein, O.; Zangerl, B.; Pearce-Kelling, S.; Sidjanin, D.J.; Kijas, J.W.; Felix, J.; Acland, G.M.; Aguirre, G.D. Linkage disequilibrium mapping in domestic dog breeds narrows the progressive rod-cone degeneration interval and identifies ancestral disease-transmitting chromosome. *Genomics* **2006**, *88*, 541-550, doi:10.1016/j.ygeno.2006.05.013.
44. Santos-Anderson, R.M.; Tso, M.; Wolf, E.D. An inherited retinopathy in collies: A light and electron microscopic study. *Invest Ophthalmol Vis Sci* **1980**, *19*, 1282-1294.

45. Kukekova, A.V.; Goldstein, O.; Johnson, J.L.; Richardson, M.A.; Pearce-Kelling, S.E.; Swaroop, A.; Friedman, J.S.; Aguirre, G.D.; Acland, G.M. Canine RD3 mutation establishes rod-cone dysplasia type 2 (rcd2) as ortholog of human and murine rd3. *Mamm Genome* **2009**, *20*, 109-123.
46. Hitti, R.J.; Oliver, J.A.C.; Schofield, E.C.; Bauer, A.; Kaukonen, M.; Forman, O.P.; Leeb, T.; Lohi, H.; Burmeister, L.M.; Sargan, D., et al. Whole Genome Sequencing of Giant Schnauzer Dogs with Progressive Retinal Atrophy Establishes NECAP1 as a Novel Candidate Gene for Retinal Degeneration. *Genes (Basel)* **2019**, *10*, doi:10.3390/genes10050385.
47. Murgiano, L.; Becker, D.; Torjman, D.; Niggel, J.K.; Milano, A.; Cullen, C.; Feng, R.; Wang, F.; Jagannathan, V.; Pearce-Kelling, S., et al. Complex Structural PPT1 Variant Associated with Non-syndromic Canine Retinal Degeneration. *G3 (Bethesda)* **2019**, *9*, 425-437, doi:10.1534/g3.118.200859.
48. Downs, L.M.; Wallin-Hakansson, B.; Boursnell, M.; Marklund, S.; Hedhammar, A.; Truve, K.; Hubinette, L.; Lindblad-Toh, K.; Bergstrom, T.; Mellersh, C.S. A frameshift mutation in golden retriever dogs with progressive retinal atrophy endorses SLC4A3 as a candidate gene for human retinal degenerations. *PLoS One* **2011**, *6*, e21452, doi:10.1371/journal.pone.0021452
PONE-D-11-07538 [pii].
